# Comparison of CT findings and histopathological characteristics of pulmonary cryptococcosis in immunocompetent and immunocompromised patients

**DOI:** 10.1038/s41598-022-09794-6

**Published:** 2022-04-05

**Authors:** Dong-xu Wang, Qing Zhang, Qiu-ting Wen, Guo-xu Ding, Yu-guang Wang, Feng-xia Du, Tian-yu Zhang, Xiao-yang Zheng, Hou-yi Cong, You-li Du, Jun-zhi Sang, Ming-da Wang, Shan-xin Zhang

**Affiliations:** 1 Departments of CT, the Second Affiliated Hospital of Qiqihar Medical College, Qiqihar, 161006 Heilongjiang China; 2 Center of Infectious Diseases, Beijing Ditan Hospital, Capital Medical University, No. 8 Jing Shun East Street, Beijing, 100015 China; 3 Department of Pathology, Qiqihar Medical College, Qiqihar, 161006 Heilongjiang China; 4 Party Committee Office, the Second Affiliated Hospital of Qiqihar Medical College, Qiqihar, 161006 Heilongjiang China; 5 Department of Microbiology, Qiqihar Medical College, Qiqihar, 161006 Heilongjiang China

**Keywords:** Respiratory tract diseases, Fungal infection

## Abstract

Pulmonary cryptococcosis (PC) is a common fungal infectious disease, and infection can occur in patients with any immune function. To better understand PC, we compared the CT findings and histopathological results in immunocompetent and immunocompromised patients. The clinical data of 68 patients with PC were collected retrospectively and divided into the immunocompetent group and immunocompromised group. The clinical characteristics, CT manifestations and histopathological characteristics of the two groups of patients were compared. Forty-two patients (61.8%) were immunocompetent, and 26 patients (38.2%) were immunocompromised. Compared with immunocompromised patients, 57.14% (24/42) of immunocompetent patients were asymptomatic (*p* = 0.002). Compared with immunocompetent patients, cough (14/26, 53.9%) and fever (13/26, 50.0%) were the main symptoms in immunocompromised patients *(p* = 0.044, *p* = 0.007). Nodular lesions (97.6%, 41/42) were the most common CT type in immunocompetent patients, and the CT characteristic was a single lesion (25/42, 59.5%); the main histopathological type was nodular fibrogranuloma (30/42, 71.4%), and the main histopathological characteristic was inflammatory granuloma (31/42, 73.81%) formed by macrophage phagocytosis of Cryptococcus. Consolidation (15/26, 57.7%) was more common in the CT type of immunocompromised patients. Multiple lesions (24/26, 92.31%), air bronchial signs (19/26, 73.081%) and cavities (9/26, 34.62%) were the main CT characteristics. The mucinous colloid type (19/26, 73.1%) was its main histopathological type, which was mainly characterized by a small amount of surrounding inflammatory cell infiltration (17/26, 65.4%). There were significant differences in the classification and characteristics of CT and pathology between the two groups (*p* < 0.05). Through the CT manifestations and histopathological characteristics of PC under different immune function states, it was found that immune function has a significant impact on the CT manifestations and histopathological characteristics of patients with PC.

## Introduction

Any tissue and organ of the human body can be infected by Cryptococcus, and the most common infection is in the respiratory system, followed by the central nervous system and skin^1,2^. PC is a pulmonary fungal disease caused by cryptococcal infection^3^. A retrospective survey of pulmonary mycosis in China showed that PC ranked third^4^, and most of the patients had normal immune function. The incidence rate of cryptococcal infection in immunocompromised patients is approximately 6–10%^5^. Cryptococcal spores are dormant or disseminated in the human body due to the different immune statuses of patients, resulting in different clinical characteristics^6^.

Previous studies on immunocompromised PC were mainly conducted in AIDS patients^7^. A recent document found^8^ the clinical and CT characteristics of PC patients with different immune states but did not describe the histopathological characteristics. Few studies have proposed using histopathological characteristics to explain the CT manifestations of PC patients in different immune states.

In this study, we retrospectively investigated the clinical data of 68 patients with PC. By comparing the relationship between CT manifestations, histopathological characteristics and immune function, we studied the clinical symptoms, CT manifestations and histopathological characteristics of PC under different immune functions.

## Materials and methods

### Patients

We retrospectively analyzed the medical records of 68 patients with PC from January 2015 to December 2020. All patients were histopathologically confirmed to have pulmonary cryptococcosis. Among them, 30 patients underwent pulmonary segment or wedge resection, 25 underwent CT-guided percutaneous needle biopsy, 11 underwent bronchoscopy biopsy, and 2 underwent transbronchial needle biopsy under the guidance of ultrasound bronchoscopy. The histopathological specimens were fixed with formalin, embedded in paraffin, sectioned at 4 mm, and then stained with HE and PAS. Two experienced pathologists analyzed all histopathological sections and divided them into three types according to histopathological diagnostic criteria^9,10^. Mucinous colloid type (composed of cryptococcus, large necrotic and mucinous degenerative connective tissue), inflammatory granuloma type (granuloma mainly infiltrated by inflammatory cells, accompanied by proliferative inflammation of fibroblasts and fibrous tissue), nodular fibrogranulomatous (nodular fibrous granuloma formed by phagocytosis of Cryptococcus by macrophages). The histopathological features were recorded (inflammatory granuloma formed by phagocytosis of Cryptococcus by macrophages, nongranulomatous lesions with a large number of Cryptococcus free in the alveolar cavity, a large number of inflammatory cells infiltrating fibrous tissue proliferating, and a small amount of inflammatory cell infiltration).

### Chest CT

The patient underwent a 64-slice CT scan. The patient lay supine on the scanning table. Breath-hold scanned after inhalation. The voltage was 120 kV, the current was 180 mA, the layer thickness was 1 mm, and the layer distance was 1 mm. Two experienced radiologists analyzed all CT images according to the following imaging diagnostic criteria. A lesion with a clear boundary was defined as a nodule. Lesions with unclear borders were defined as consolidative. Diffuse centrilobular micronodule infiltration was defined as a miliary pattern. Mediastinal lymphadenopathy was defined as multiple enlarged lymph nodes in the mediastinum. The predominant CT patterns were divided into four types: nodular, consolidation, miliary pattern and mediastinal lymphadenopathy. At the same time, the characteristics of CT (number of lesions, air bronchial sign, halo sign, cavitation, calcification and fibrous stripes) were also recorded.

### Ethical approval and informed consent

This study was conducted in accordance with the amended Declaration of Helsinki. The study protocol was approved by the Ethics Committee of the Second Affiliated Hospital of Qiqihar Medical College. As it was a retrospective study, the ethics committee of the Second Affiliated Hospital of Qiqihar Medical College decided that informed consent of the patient was not required. Patients' names needed to be hidden during the study.

### Group classification

According to the immune status, the patients were divided into immunocompetent and immunocompromised groups. The immunocompromised group needs to meet one of the following medical histories: long-term treatment with immunosuppressive drugs or corticosteroids, severe diabetes with associated organ damage, malignant tumor (under immunosuppressive chemotherapy), HIV infection, decompensated liver cirrhosis, organ transplantation or other systemic diseases (such as systemic lupus erythematosus). At the same time, one of the following conditions was met: serum immunoglobulin IgG (7–16 g/L), IgA (0.4–2.3 g/L) or IgM (0.7–4.0 g/L); any two values were below the lower limit of the normal range; neutrophil count in peripheral blood was below 2.0 × 10^9^ cells/L (Neutropenia) or lymphocyte count was below 1,000 cells/μL (Lymphopenia); the percentage of CD3 cells in peripheral blood was less than 62%, the percentage of CD4 cells was less than 30%, or the ratio of CD4 to CD8 was less than 1.5.

### Statistical analysis

Statistical analyses were performed using SPSS 18.0 software. Measurement data are expressed as the mean ± standard deviation, the independent sample t-test (normal distribution) was used, and the Mann–Whitney test (nonnormal distribution) was adopted. Categorical variable data were tested for significance using χ2 or Fisher’s exact test. A P value < 0.05 was considered to indicate statistical significance.

### Ethics approval and consent to participate

The study protocol was approved by the Ethics Committee of the Second Affiliated Hospital of Qiqihar Medical College (Qiqihar, 161006, China).

## Results

### Demographics and clinical data

Among 68 patients with PC, 66.2% (45/68) were male, and 33.8% (23/68) were female. The average age was 47.58 (range 3–76) years. A total of 8.8% (6/68) of the patients had a clear history of exposure. Among them, 4 patients raised chickens and pigeons at home, 1 was a cook who had contact with live poultry before the onset of illness, and 1 patient worked in a live poultry market. A total of 61.8% (42/68) were immunocompetent patients. A total of 38.2% (26/68) of patients were in the immunocompromised group. Among them, 7 patients had malignant tumors and were receiving immunosuppressive chemotherapy, and the percentage of CD3 cells in peripheral blood was less than 62%, or the percentage of CD4 cells was less than 30%, or the ratio of CD4 to CD8 was less than 1.5; 7 patients were being treated with corticosteroids, and the neutrophil count in peripheral blood was below 2.0 × 10^9^ cells/L or the lymphocyte count was below 1000 cells/µL; 4 patients had severe diabetes with organ damage, and the percentage of CD3 cells in peripheral blood was less than 62%, or the percentage of CD4 cells was less than 30%, or the ratio of CD4 to CD8 was less than 1.5; 3 patients had decompensated liver cirrhosis and serum immunoglobulin IgG (7–16 g/L), IgA (0.4–2.3 g/L) or IgM (0.7–4.0 g/L), any two of which were below the lower limit of the normal range; 3 patients had AIDS, and the percentage of CD4 cells was less than 30%; and 2 patients were treated with immunosuppressive drugs for a long time, and the neutrophil count in peripheral blood was below 2.0 × 10^9^ cells/L.

There was no significant difference in age between the two groups of patients (*p* = 0.264). A total of 57.1% (24/42) and 57.1% (24/42) of immunocompetent patients were asymptomatic, and they were discovered accidentally through chest radiographs or low-dose CT scans. Eighty-eight percent (21/26) of immunocompromised patients were symptomatic. The difference between the two groups was statistically significant (*p* = 0.002). Cough (38.2%, 26/68) and fever (50.0%, 13/26) were more likely to occur in immunocompromised patients, and there was statistical significance between the two groups (*p* = 0.044, *p* = 0.007) (Table 1).

**Table 1:** Comparison of demographics and clinical symptoms of patients with PC in the immunocompetent group and immunocompromised group.

	Immunocompetent (*n* = 42)	Immunocompromised (n = 26)	*p* Value
Male	32 (76.19)	13 (50.00)	0.027
Age, year	49.29 ± 12.90	45.00 ± 18.57	0.264
Asymptomatic	24 (57.14)	5 (19.23)	0.002
Cough	12 (28.57)	14 (53.85)	0.044
Fever	8 (19.05)	13 (50.00)	0.007
Chest pain	3 (7.14)	6 (23.08)	0.129

### CT manifestations

**Figure 1:**
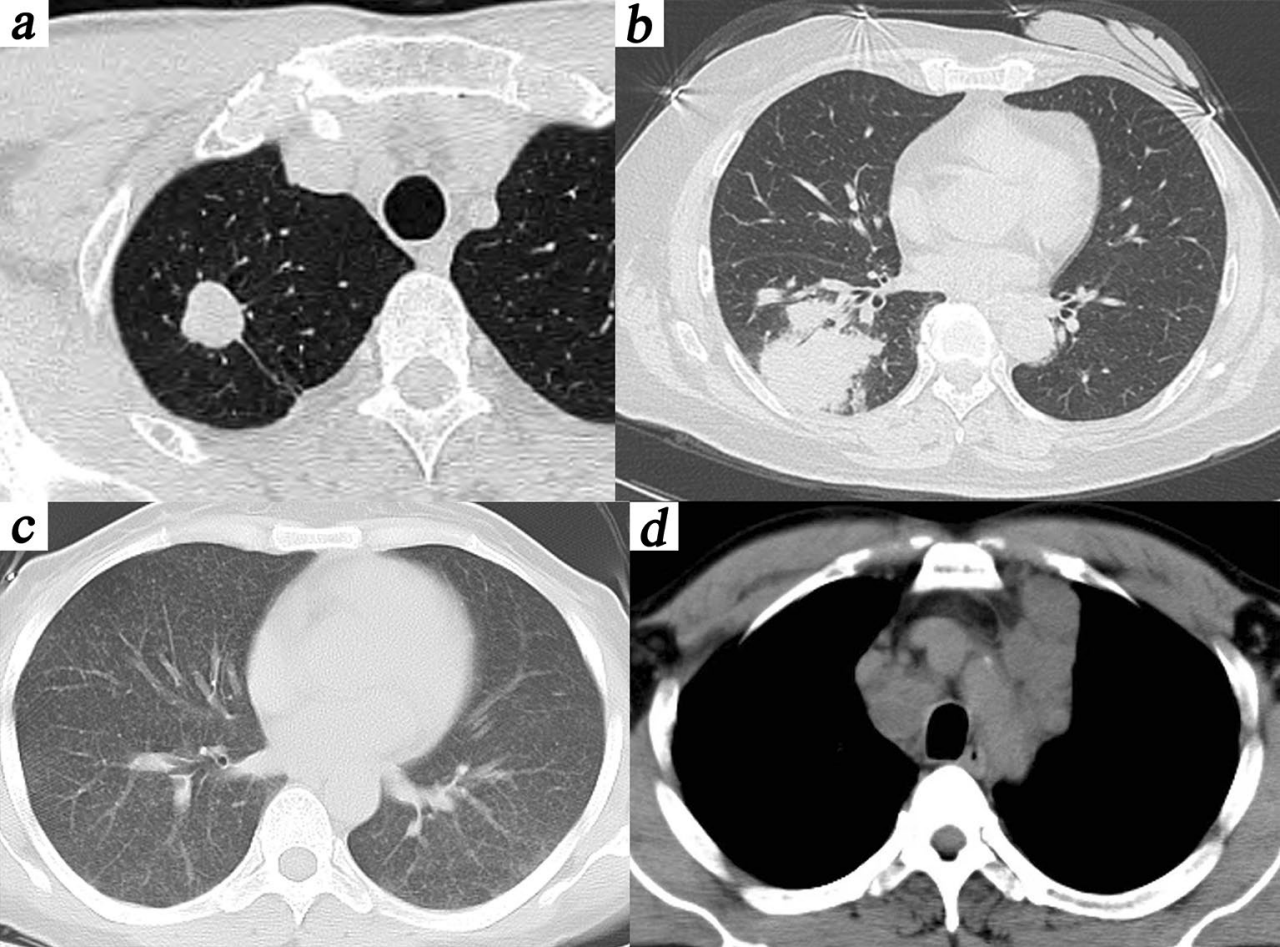
The CT manifestations of PC patients: (**a**) A 41-year-old male with normal immune function indexes was asymptomatic. Nodular type: A single nodule with smooth margins and uniform density was seen in the upper lobe of the right lung. (**b**) A 58-year-old female, after breast cancer surgery, was infected with Cryptococcus in the lungs during chemotherapy, with symptoms of fever and cough. Consolidation and air bronchial signs were seen in the right lower lobe. (**c**) A 38-year-old female presented with a pulmonary infection of Cryptococcus during corticosteroid therapy, with fever and cough. Miliary pattern: Multiple miliary nodules were seen in both lungs. (**d**) A 50-year-old male was immunocompromised and had cough. Mediastinal lymphadenopathy was seen in her lung CT.

According to the CT classification of the two groups, the immunocompetent patients were mainly nodular (59.5%, 25/42) (Fig. 1a), and the immunocompromised patients were mainly consolidated (57.7%, 15/26) (Fig. 1b). There was statistical significance between the two groups (p < 0.001). The difference between miliary pattern (Fig. 1c) and mediastinal lymphadenopathy (Fig. 1d) was not statistically significant. (p = 0.143). A single lesion (59.5%, 25/42) was the main CT characteristic of immunocompetent patients. The immunocompromised patients had CT characteristics of multiple lesions (92.3%, 24/26), air bronchial signs (73.1%, 19/26) and cavitation (34.6%, 9/26) (Table 2).

**Table 2:** Comparison of CT findings of patients with PC in the immunocompetent group and immunocompromised group.

CT manifestations	Immunocompetent (n = 42)	Immunocompromised (n = 26)	*p* Value
**CT type**			
Nodular	41 (97.6)	7 (26.9)	< 0.001
Consolidation	1 (2.4)	15 (57.7)	< 0.001
Miliary pattern	0 (0.00)	2 (7.7)	0.143
Mediastinal lymphadenopathy	0 (0.00)	2 (7.7)	0.143
**CT characteristic**			
Sigle lesion	25 (59.5)	2 (7.7)	< 0.001
Air bronchial sign	11 (26.2)	19 (73.1)	< 0.001
Halo sign	12 (28.6)	10 (38.5)	0.397
Cavitation	5 (11.9)	9 (34.6)	0.024
Calcification	3 (7.1)	2 (7.7)	1.00
Fibrous stripes	19 (45.2)	11 (42.3)	0.813

### Histopathological results

**Figure 2:**
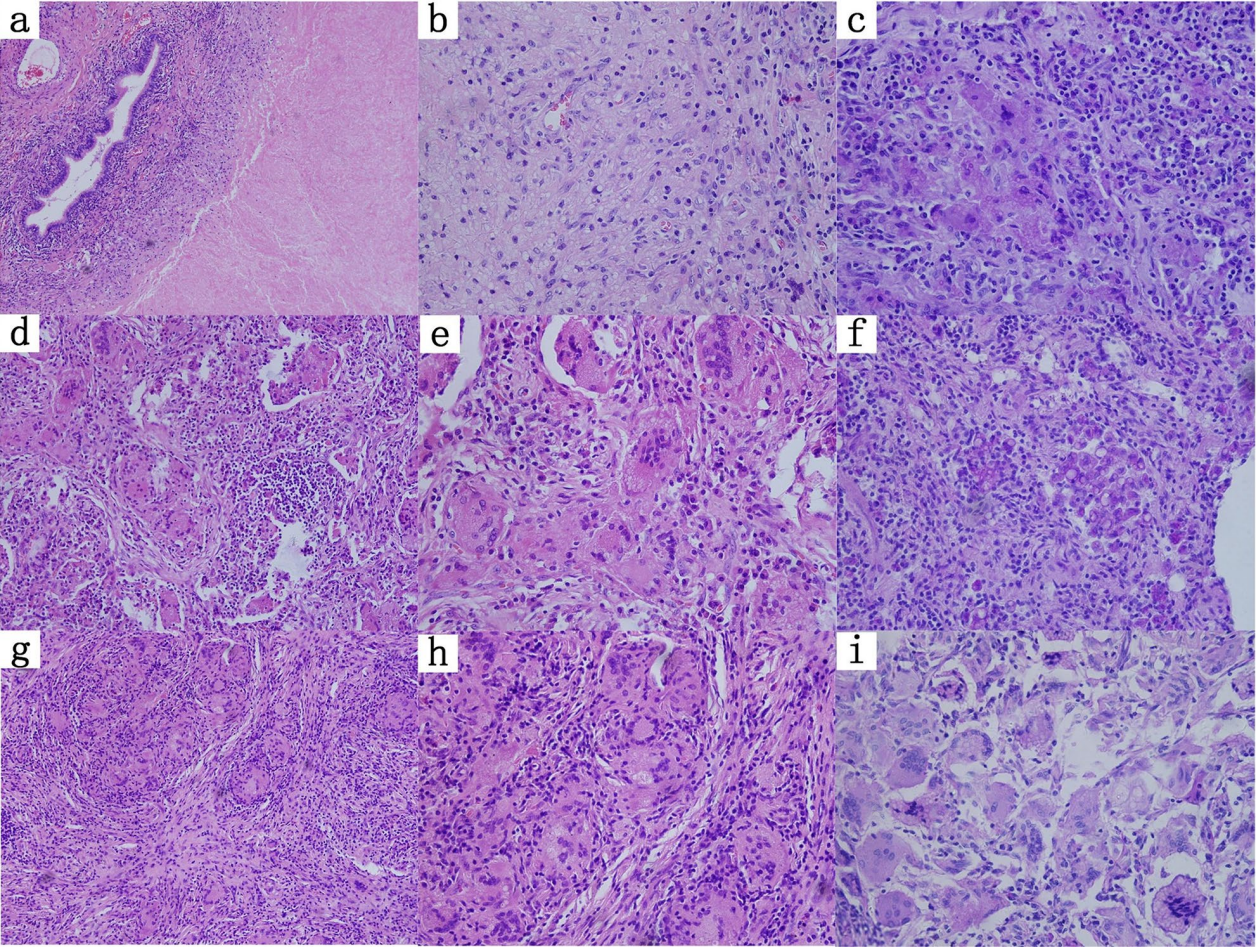
The histopathological characteristics of PC patients. Mucinous mucoid type: (**a** HE staining × 20 and **b** HE staining × 40) mainly consisted of large necrotic and mucinous connective tissue and mass cryptococci without obvious granulomatous lesions. The lung tissue around the lesion showed serous exudative inflammation. (**c** PAS staining × 40) A large number of free cryptococcal bodies were found in the alveolar cavity. Inflammatory granulomatous lesions: (**d** HE staining × 20 and **e** HE staining × 40) mainly by a large number of mononuclear macrophages, neutrophils and other inflammatory cells infiltration, inflammatory cells diffuse distribution, the edge of the lesion accompanied by a large number of fibroblast proliferation and inflammatory cell infiltration, (**f** PAS staining × 40) a small number of scattered cryptococcal bodies were found in the bronchial wall around the lesion. Nodular fibrogranulomatous lesions: (**g** HE staining × 20 and **h** HE staining × 40) nodular fibrogranuloma lesions were mainly formed by mononuclear macrophages and multinucleated giant cells, with different sizes of multinucleated giant cells. (**i** PAS staining × 40) A small amount of Cryptococcus was found in the granuloma.

There were three different types of tissue structures that could be observed under a microscope: 33.8% (23/68) of patients presented with mucocolloid lesions (Fig. 2a b c), which were a mixed composition of clusters of cryptococcal cells, large necrosis and mucinous degeneration of connective tissue. There was a small amount of inflammatory cell infiltration and fibrous tissue hyperplasia in the center of the lesion but no obvious granulomatous lesions. Inflammatory granulomatous lesions were found in 19.1% (13/68) of the patients (Fig. 2d–f) and were mainly infiltrated by inflammatory cells, such as a large number of mononuclear macrophages and neutrophils, accompanied by fibroproliferative inflammation, and inflammatory cells were diffusely distributed. A total of 47.1% (32/68) of patients developed nodular fibrogranulomatous lesions (Fig. 2g–i) composed of a mixture of mononuclear macrophages, multinucleated giant cells, and a large number of fibrous histiocytes. Nodular fibrogranulomatous lesions of different sizes and multinucleated giant cells of different sizes were present. There were 2 cases (7.7%) of nodular fibrogranuloma type in immunocompromised patients and 19 cases (73.1%) of mucinous colloid type. There were 30 cases (71.4%, 30/42) of nodular fibrogranuloma type in immunocompetent patients and 4 cases (9.5%) of mucinous colloid type, and the difference was statistically significant (p < 0.001). Macrophages in the center of the lesions phagocytosed Cryptococcus to form inflammatory granulomas, and a large amount of inflammatory cell infiltration around them was the histopathological characteristic of 31 immunocompetent patients (73.8%). No obvious granulomatous lesions in the center of the lesion, a large number of free cryptococci in the alveolar cavity, and a small amount of inflammatory cell infiltration around the lesion were the histopathological characteristics of 16 immunocompromised patients (61.5%) (Table 3).

**Table 3:** Comparison of histopathological characteristics of patients with PC in the immunocompetent group and immunocompromised group.

Histopathological results	Immunocompetent (n = 42)	Immunocompromised (n = 26)	*P* Value
**Type**			
Mucinous colloid type	4 (9.5)	19 (73.1)	< 0.001
Inflammatory granuloma type	8 (19.1)	5 (19.2)	1.00
Nodular fibrogranulomatous	30 (71.4)	2 (7.7)	< 0.001
**Characteristic**			
Non-granulomatous lesions with a large number of Cryptococcus free in the alveolar cavity	11 (26.2)	16 (61.5)	0.004
A large number of inflammatory cells infiltrated and fibrous tissue proliferated	30 (71.4)	9 (34.6)	0.003

## Discussion

Cryptococcus is commonly found in bird feces and soil^11,12^, and it appears that immunocompromised patients and people with a history of environmental exposure are more susceptible to infection. A growing body of literature now points out that it is more common in normal people with no history of exposure^13,14^. A total of 8.8% of the patients in the study had a history of environmental exposure, and 61.8% of the patients were in the normal population. This may be related to the incidental discovery of PC in healthy people. In this study, immunocompromised patients were more likely to develop symptoms, among which cough (53.9%) and fever (50.0%) were the most common. Qu et al. also pointed out that immunocompromised patients are more prone to cough and fever^15^. Immunodeficiency reduces CD4 cells, which may lead to a greater burden of Cryptococcus in patients, and systemic inflammation may cause symptoms. This view has also been mentioned by He et al.^16^.

The CT manifestations of PC are variable, and many patients are misdiagnosed by doctors. We used histopathological results to explain the CT manifestations of PC in different immune states so that clinicians can better understand the impact of the patient's immune function on PC. Min et al. reported the CT findings of 78 patients with PC, including 61 immunocompetent cases and 17 immunocompromised cases^17^. The article only analyzed the CT manifestations under different immune states without comparing the histopathological results. We divided the CT manifestations of PC into four types. Among them, nodular consolidation was the most common, and miliary patterns and mediastinal lymphadenopathy were rare. The histopathological results of PC were divided into three types: mucinous colloid type, inflammatory granuloma type and nodular fibrogranulomatous. The immune status of patients was an influencing factor of CT performance. The results of our study showed that the CT findings of PC patients with normal immune function indexes were more common in the nodular type, and the corresponding histopathological classification was the nodular fibrogranulomatous type. The mechanism was that Cryptococcus can be phagocytosed by a large number of mononuclear giant cells after entering the body of immunocompetent patients, forming tough granulomatous induration foci, making it difficult for Cryptococcus to spread and form nodules. We also found that consolidation was more common in immunocompromised patients, and its corresponding histopathology was mucinous colloid type. Under the microscope, we found that a large number of free cryptococci can be seen in the alveolar cavity, which infect multiple places in the lungs through the respiratory tract, forming exudative inflammation. This better explained why consolidation was more common in immunocompromised patients. Consolidation and multiple lesions represented the spread of infection, reflecting the difficulty of controlling fungal infections in immunocompromised patients^18^. We also observed no obvious inflammatory reaction or lymphocyte infiltration in the lesions under the microscope, which Zinck et al. mentioned in the article^19^. This study found that the number of multiple lesions, air bronchial sign and cavitation were more common in immunocompromised patients, which was consistent with the results of previous literature studies^20–22^. Xie et al. reported that air bronchial signs are common in immunocompromised patients with PC^23^. In this study, air bronchial signs were found in 73.08% of immunocompromised patients. This may be related to the fact that bronchioles are more likely to be blocked in immunocompromised patients with cryptococcal infection^24^, resulting in a clear display of distal blockage at the proximal end of the bronchi. Cavitation was found in 34.62% of immunocompromised patients. Similar results were reported in previous articles^25,26^. Histopathological evidence was found in our study to explain this phenomenon. We observed under the microscope that the lung tissue had different degrees of exudative inflammation. The necrotic lung tissue with mucoid gelatinous degeneration was drained through the drainage bronchus, and cavities were formed on CT images, which was the reason why cavities were more likely to appear in immunocompromised patients.

Cryptococcal culture is one of the methods for the diagnosis of PC, but the clinical detection rate is low^27^. Histopathological examination became the most accurate method for the diagnosis of PC in our clinical work. We observed by histopathological HE staining that Cryptococcus sometimes showed light blue or nonstained round vacuolar bodies of various sizes, with a ring-shaped translucent halo (colloid capsule) around it. If not carefully observed, it is easy to miss a diagnosis. The microscopic morphology of these cryptococci was consistent with the observation results of Wang et al.^28^.

The definition of immunocompromised as defined was limited in our study. There are currently no accepted diagnostic criteria for the definition of immunocompromised. Previous studies^11,29–32^ have shown that neutrophils and lymphocytes have anti-inflammatory effects and can enhance the body's immunity by promoting the proliferation of T cells. Immunoglobulin has the function of regulating immune function, and CD3 and CD4 cells are the reactive cells of the immune response and can affect the production of B cell antibodies. CD8 is a marker of suppressor T cells, and a decrease in CD4/CD8 indicates poor immunity. In patients with underlying diseases, when the above indicators are reduced, we consider it immunocompromised.

## Conclusions

By comparing the CT manifestations and histopathological data of immunocompetent and immunocompromised patients with PC, we found that the immune function status had a significant impact on CT manifestations and histopathological results.

## Abbreviations

PC: Pulmonary cryptococcosis
AIDS: Acquired immunodeficiency syndrome
CT: Computerized tomography
HE: Hematoxylin–eosin
PAS: Periodic acid-Schif
